# Attenuation of postoperative adhesions using a modeled manual therapy

**DOI:** 10.1371/journal.pone.0178407

**Published:** 2017-06-02

**Authors:** Geoffrey M. Bove, Susan L. Chapelle, Katherine E. Hanlon, Michael P. Diamond, David J. Mokler

**Affiliations:** 1 University of New England College of Osteopathic Medicine, Department of Biomedical Sciences, Stella Maris 102 Biddeford ME, United States of America; 2 Squamish Integrated Health, Cleveland.Squamish, BC, CANADA; 3 Medical College of Georgia, Augusta University, Department of Obstetrics and Gynecology, Augusta, GA United States of America; University of Florida, UNITED STATES

## Abstract

Postoperative adhesions are pathological attachments that develop between abdominopelvic structures following surgery. Considered unavoidable and ubiquitous, postoperative adhesions lead to bowel obstructions, infertility, pain, and reoperations. As such, they represent a substantial health care challenge. Despite over a century of research, no preventive treatment exists. We hypothesized that postoperative adhesions develop from a lack of movement of the abdominopelvic organs in the immediate postoperative period while rendered immobile by surgery and opiates, and tested whether manual therapy would prevent their development. In a modified rat cecal abrasion model, rats were allocated to receive treatment with manual therapy or not, and their resulting adhesions were quantified. We also characterized macrophage phenotype. In separate experiments we tested the safety of the treatment on a strictureplasty model, and also the efficacy of the treatment following adhesiolysis. We show that the treatment led to reduced frequency and size of cohesive adhesions, but not other types of adhesions, such as those involving intraperitoneal fatty structures. This effect was associated with a delay in the appearance of trophic macrophages. The treatment did not inhibit healing or induce undesirable complications following strictureplasty. Our results support that that maintained movements of damaged structures in the immediate postoperative period has potential to act as an effective preventive for attenuating cohesive postoperative adhesion development. Our findings lay the groundwork for further research, including mechanical and pharmacologic approaches to maintain movements during healing.

## Introduction

Adhesions between structures that are normally free-moving are a ubiquitous side effect of abdominal and pelvic surgeries. Although most postoperative adhesions are asymptomatic, they are a leading cause of small bowel obstruction, infertility, and repeat surgeries, and have been implicated in chronic pelvic pain [1–8]. Although symptomatic postoperative adhesions can be treated by reoperation, the outcomes are not always successful, and it is generally agreed that preventing postoperative adhesions is preferable [9–15]. There have been numerous preventive approaches, including limiting peritoneal damage and placing a liquid or solid barrier between the structures. Since none are consistently effective [16, 17], the prevention of postoperative adhesions remains elusive.

A less researched approach to prevent postoperative adhesions has been to maintain movement of the structures while healing. Surgery inhibits normal intestinal movements, referred to as postoperative ileus, and movements are also reduced by opiates [18, 19]. In humans, impaired postoperative motility is considered normal, lasting on average 1–2 days for the small intestine and 2–3 days for the colon [20], but often lasts much longer [18]. It is possible that this stasis facilitates adhesion development, since the key epoch for adhesion formation is during the first 3 postoperative days [14]. Theoretically, if the damaged surfaces were periodically moved during this critical time period, passively or actively, adhesion development would be limited. This possible etiological link was discussed more than a century ago (see references in [21] and [22]), and two studies showing efficacy of prokinetic agents (neostigmine) were published more than 50 years ago [22, 23]. The topic received little further consideration, perhaps because such agents are not well tolerated by patients following surgery. Our study builds upon these concepts by offering a potentially more tolerable treatment option, as well as a feasible model for further study (rats versus larger mammals).

Extending the hypothesis that maintained movements would inhibit postoperative adhesions, we adapted a manual therapy method called “visceral manipulation” and applied it in a model of postoperative adhesions in rats. In clinical practice, therapists simultaneously palpate and mobilize the abdominal contents, imparting passive movements between the abdominopelvic structures. This has been shown to have clinical efficacy for patients with constipation [24, 25], indicating that it is prokinetic. We used a massage therapist (SLC) with 20 years of clinical experience using the technique referred to in the field as visceral manipulation to scale this treatment approach for a rat, and called the treatment “modeled manual therapy” (MMT). Our earlier studies showed that our therapist was able to detect and disrupt cohesive postoperative adhesions, and that the treatment also reduced postoperative ileus [26, 27]. In this manuscript we report the effects of MMT for the prevention of postoperative adhesions, and investigate a possible mechanistic link between MMT and alteration in the adhesion development cascade, beginning with macrophages.

## Methods

### Animals and groups

All procedures were consistent with the Guide for the Care and Use of Laboratory Animals (National Research Council, USA), and were approved by the University of New England Institutional Animal Care and Use Committee (Animal Welfare Assurance Number D16-00277). All surgeries were performed under isoflurane anesthesia, and all efforts were made to minimize suffering.

A total of 147 rats were used for the described experiments (Table 1). One hundred thirty one female SD rats were obtained from Charles River Laboratories (USA), and weighed 190–210 g when used. These rats were placed into 12 groups to study the effect of MMT on postoperative adhesions. All rats were handled for 3–4 minutes by one of the authors on the 3 days prior to the surgeries, and were accommodated to enclosures that allowed monitoring of their fecal output [28]. Rats were operated upon to induce adhesions, and assigned to receive treatment or remain untreated. Thirteen rats were used for macrophage harvesting only. An additional 16 male SD rats weighing 350–450 g were obtained for a sub-study of the safety of MMT following strictureplasty. These rats were placed in 2 groups of 7 (treated and untreated).

**Table 1:** Experimental groups.

Procedure	Survival Days	Untreated (losses)	Treated (losses)	Total (losses)
None	0 (Control)	13	0	13
Cecal Hinge	1	7	7	14
Cecal Hinge	2	7 (1*)	7	14 (1)
Cecal Hinge	4	7	9 (2*)	16 (2)
Cecal Hinge	7	8	13 (3*)	20 (3)
Cecal Hinge MD**	7	14 (2*)	8 (2*, 1#)	22 (5)
Adhesiolysis	28 / 7	15 (3#)	15	30 (3)
Strictureplasty	7	7+2 ##	7	18
	Totals (losses)	80 (6)	66 (8)	147 (14)

* One of the stitches avulsed; area data were not included.

** A separate experiment was performed using multiple doses (MD) of pain medication.

#Postsurgical complications led to removal of these rats from the study.

## Two normal rats were used for baseline measurements.

### Surgical details and experimental protocols

#### Experiment 1: Modeled manual therapy for attenuation of postoperative adhesions

**Fig 1:**
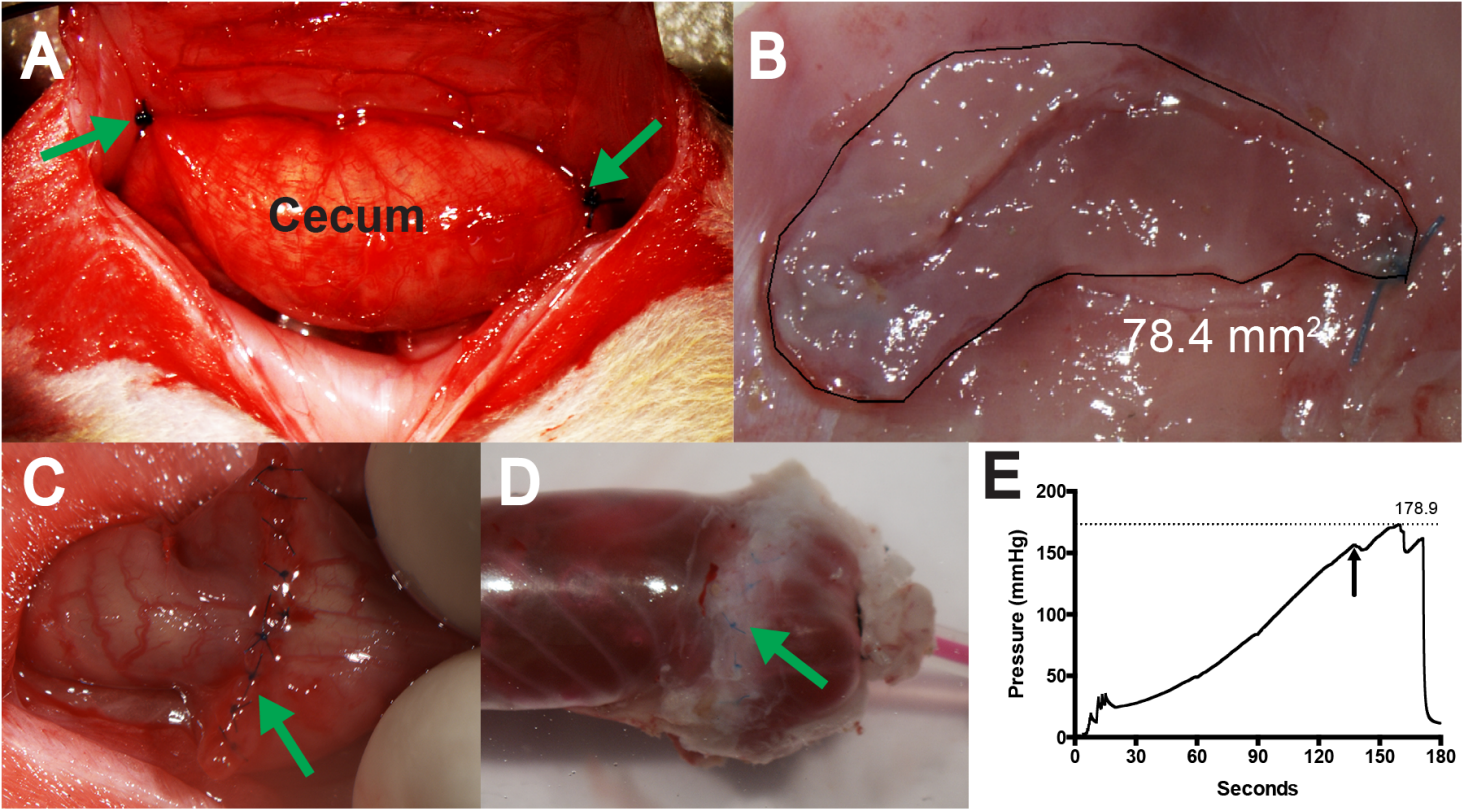
Experimental methods. A. To create the cecal hinge, the abraded cecum was stitched in two places (arrows) to appose a 1 cm X 2 cm area where the peritoneum had been removed. B. Quantification of the primary adhesion. The free cecum was cut from the adhesion area, the contents removed, and the edges trimmed. The adhesion was outlined and the area recorded. C. Completed strictureplasty surgery. D. The intestinal segment 7 days following strictureplasty was instrumented and inflated until suture failure. The sutures can be seen beneath the fatpad adhesion that encased the suture line. E. Sample trace of intraluminal pressure. The first dip (arrow) was associated with mesothelial splitting at a different site from the suture line. This sample withstood a pressure of 179 mmHg.

Our hypothesis was that adhesions form secondary to postoperative continued adjacency of injured surfaces (e.g. cecum, intestines, and abdominal wall) and that externally applied manipulations during the critical first days after surgery could dynamically alter the positioning of these structures, thus reducing or preventing adhesion formation. We developed a modified cecal-sidewall model to limit but not fully prevent peristalsis or naturally occurring movements of the cecum. All surgeries were performed by the same surgeon (GMB). Following anesthesia with isoflurane (1.75–2.25% in pure oxygen) and appropriate surgical preparation, the abdominal wall was incised just right of the linea alba for 2.5 cm. Using atraumatic forceps, the greater omentum was displaced cranially if covering the intestines. The cecum and 5–6 cm of small intestine were exteriorized. The cecum was grasped with gloved fingers, and a sterile toothbrush with a 13 mm circular head (Braun, USA) was stroked ~8 times over the entire cecum, causing destruction of the mesothelium (indicated by strands of tissue forming on the brush) and inducing multiple petechial hemorrhages over the entire cecum without frank bleeding (Fig 1A). The small intestine and cecum were then placed back in the abdominal cavity in their normal anatomical position.

A loop of suture was used to retract the abdominal wall over a custom brace, revealing a 2.5 cm long and 1 cm wide area. A 2 cm long X 1 cm wide rectangle was outlined with shallow scalpel cuts guided by calipers. The peritoneum was undermined using spring scissors, avoiding the superior epigastric artery. The flap was retracted medially to include part of the transversus abdominis muscle, and cut at the previously marked line, leaving a 2 cm X 1 cm area of scarified muscle. The cecum was retrieved and two 4–0 nylon sutures were placed attaching the greater curvature to the abdominal wall, 1–2 mm cranial and caudal to the centerline of the abdominal wall lesion, with no tension between the sutures (Fig 1A). Because the cecum was free to move along an axis between the sutures, we termed this the “cecal hinge” model [29]. We termed the resultant adhesion development at this location the “primary adhesion.”

The abdominal wall was closed in layers using interrupted 4–0 nylon sutures. In the primary experiment, rats were given one dose of buprenorphine (0.05 mg/kg sc) and placed in individual custom enclosures to recover. Rats usually resumed their normal activity levels within one hour. Fecal pellet discharge was monitored and reported as pellets per 4-hour epoch, over 24 hours. We previously reported that prolonged dosage of buprenorphine leads to more severe reductions in transit time and fecal pellet discharge [28], suggesting reduced movements of the structures. Therefore, we also tested whether prolonged buprenorphine administration caused more extensive adhesion formation by performing an experiment using 3 doses of buprenorphine with a reported efficacy of 36 hours (0.05 mg/kg i.p. q12).

Rats were treated using MMT as previously described [26, 27]. The treatment was designed and performed by a professional massage therapist with >20 years clinical experience, and was intended to mobilize the entire gastrointestinal tract and encourage intraperitoneal fluid movement between the peritoneal surfaces. A sample treatment can be seen in Supporting Information (S1 Video; consent was obtained to use this video). Treatments were performed immediately after the surgery and every 4 hours thereafter other than between 22:00 and 07:00. Treatments were performed until the planned end-point, or for a maximum of 4 days. Control rats were not handled after surgery because picking up and holding active rats involves gravitational forces and deflection of the abdomen. This was perceived as a possible confound.

After 1, 2, 4, or 7 days (random assignment), rats were given an overdose of isoflurane and exsanguinated by opening the thorax and lesioning the heart. This prevented bleeding during the necropsy, which we previously noted interfered with the rating methods and also with the isolation of macrophages. The abdominal skin was reflected distally and the abdominal wall was opened just distal to the xyphoid. To collect macrophages, 10 ml of sterile buffer was injected into the intraperitoneal space just distal to the xyphoid prior to adhesion evaluation. After gentle mobilization, a flexible plastic feeding tube with a small polyester wool bonnet was then carefully guided laterally into the paraspinal gutters, avoiding the adhesion area, and the lavage fluid was aspirated (8.5–9 ml). Using a surgical microscope at 3.75X, a wide oval of the abdominal wall was excised but left in place to isolate but not disrupt the primary adhesion.

A necropsy was performed and recorded for later analysis using a digital camera (Nikon DS-Fi1, USA) controlled by Nikon Elements software. The surgeon (GMB) systematically examined the abdomen, identifying and then disrupting all adhesions. The necropsy proceeded by examining adhesions involving the greater omentum, the left and right adnexal fat pads, and then any other adhesions between the cecum and other organs including the cecum to itself. The primary adhesion was not disrupted during this process. After the necropsy video was finished, the abdominal wall and adhered portion of the cecum were removed *en bloc*.

#### Evaluation of the primary adhesion

The adhered part of the cecum and the abdominal wall panel were placed cecum-up on a pad. If both stitches were not patent, the data for the primary adhesion was excluded. Under microscopic guidance, the cecum contents were removed with cotton swabs, and the edges of the patch of cecum were trimmed carefully to reveal the boundaries of the adhesion. A calibrated photograph was taken, and the adhesion area measured using Nikon Elements (Fig 1B).

#### Intraperitoneal adhesion rating

The severities of the intraperitoneal adhesions (not including the primary adhesions) were rated using a consensus approach that we have recently described [29]. Four investigators familiar with the methods but not otherwise involved in the experiments performed the ratings.

#### Intestinal function

Part of our hypothesis included that post-operative bowel stasis (ileus) could be at least in part causative of postoperative adhesion formation. We evaluated postoperative ileus by observing fecal discharge, using methods previously described [28]. Discharge over 4 hour epochs was chosen as the outcome measure. Pellets discharged during treatment were placed into the chambers for inclusion in counting.

#### Macrophage phenotype

We performed flow cytometry to identify relative levels of intraperitoneal M1 (inflammatory) and M2 (trophic) macrophage phenotypes using surface and intracellular markers as previously described [30]. We defined M1 macrophages as displaying increased expression of CD86 and iNOS relative to intraperitoneal macrophages isolated from naïve rats. We defined M2 macrophages as displaying increased expression of CD163 and arginase relative to intraperitoneal macrophages isolated from naïve rats. Primary antibodies included: Pacific Blue monoclonal mouse anti- CD11b/OX-42 [31] (AbD Serotec catalog # MCA275PB; RRID: AB_566459), FITC monoclonal mouse anti- HIS48 [32] (BD Biosciences catalog # 554907; RRID: AB_395595), biotin monoclonal mouse anti- CD86 [33] (Biolegend catalog # 200303; RRID: AB_313852), PE monoclonal mouse anti- CD43[34] (Biolegend catalog #202812; RRID: AB_10642816), unconjugated polyclonal goat anti- CD163 [35] (Santa Cruz Biotechnology catalog # SC-18796; RRID: AB_2291274), unconjugated polyclonal rabbit anti- iNOS [36] (Santa Cruz Biotechnology catalog # SC-649, RRID: AB_631833), and unconjugated polyclonal sheep anti- arginase1 [37] (Novus Biologicals catalog # AF5868; RRID: AB_1964500). All primary antibodies were stained at a concentration of 2 μL/10^6 cells; references listed indicate antibody validation studies. Secondary antibodies included: Alexa Fluor 647 polyclonal donkey anti-sheep IgG (Thermo-Fisher Scientific catalog # A-21448; RRID: AB_2535865), PerCP-Cy5.5 polyclonal donkey anti-goat IgG (Santa Cruz Biotechnology catalog # SC-45102; RRID: AB_1563986), and V500 conjugated streptavidin (BD Biosciences catalog # 561419; RRID: AB_10611863). All secondary antibodies were tested for non-specificity by staining rat tissue in the absence of primary antibody and measuring fluorescence. Secondary antibodies fluorescing without a primary antibody present were not used. All secondary antibodies were stained at a concentration of 0.5 μL/10^6^ cells.

#### Experiment 2. Modeled manual therapy following adhesiolysis

In 30 rats, we created adhesions using a cecal-sidewall model, followed by adhesiolysis after 28 days. Using the same methods as described above, the cecum was abraded and the left abdominal wall was lesioned. The cecum was sutured to the abdominal wall just outside the 4 corners of the abdominal wall lesion. After 28 days the rats underwent adhesiolysis surgery. The primary adhesion was sharply dissected using a surgical microscope, primarily using Castroviejo scissors. The entire gut was searched for adhesions, which were peeled apart or cut with scissors. After closure, rats were either left untreated or were treated as described above. After 7 days, the adhesions were assayed as described above.

#### Experiment 3. Modeled manual therapy following strictureplasty

To test the safety of the treatment on delicate intestinal sutures we used a model of strictureplasty. Following anesthesia and surgical preparation, a 1 cm midline incision was made and approximately 2 cm of the proximal large intestine was located and exteriorized. A blunt needle was inserted through the mesentery to hold the loop out of the abdominal cavity. A 1 cm longitudinal incision was made through the wall of the bowel. The incision was then closed using interrupted 8–0 prolene sutures (Fig 1C). The incision was copiously rinsed with sterile buffer, the intestine replaced in the abdominal cavity, and the abdominal wall closed in layers. Rats were injected with buprenorphine 0.05 mg/kg ip and placed in a clean cage for recovery. 7 rats received no treatment, and 7 rats received treatment as outlined above.

One week following surgery, rats were terminally anesthetized with isoflurane, killed by exsanguination, and a 3–4 cm section of the colon, with the strictureplasty centered, was removed and placed in buffer at room temperature. One end of the colon section was intubated with a catheter connected to an infusion pump that delivered 300 ul/min SIF containing a fluorescent red dye (Fig 1D). The other end was intubated with a catheter connected to a calibrated pressure transducer and bridge amplifier (CB Sciences), which were connected to an analog–digital converter (CED Power 1401) and data acquisition software (CED Spike 2). The infusion pump was started and pressure sampled at 100 Hz, while under visual observation through a microscope. The maximum pressure was obtained by inspection of the data trace (Fig 1E). Testing of 2 normal colons showed peritoneal splitting at 80–90 mm Hg, which was considered beyond physiological pressures.

### Data analysis

Data were compiled in Excel, and analyzed using GraphPad/Prism. Primary adhesion areas were subjected to a 2-way ANOVA corrected for multiple comparisons using Sidak post hoc tests. In the experiment using 3 doses of buprenorphine, primary adhesion areas were compared using a student’s t-test. The adhesion areas surrounding the stitches were typically 10 mm^2^ each or less. This area was included in the main statistical analysis of the areas. In many cases, the only adherent areas were at the stitches, which was essentially no adhesion. Therefore, to analyze the proportions of rats with or without adhesions, we defined total areas of less than 20 mm^2^ as “no adhesion,” and performed a Fisher’s Exact test for proportions (two-tailed). The video ratings were analyzed as previously described [29]. Rating data, fecal pellet counts, and macrophage data were analyzed using 2-way ANOVA corrected for multiple comparisons using Sidak post hoc tests. Strictureplasty results were compared using a t-test. Significance was set at p ≤ 0.05. All data are archived and accessible at the following URL: http://dune.une.edu/biomed_facpubs/20/.

## Results

### Experiment 1: Modeled manual therapy for attenuation of postoperative adhesions

Of 147 rats, 146 recovered from the surgery without incident. One untreated rat was found dead 2 days postoperatively. The treated rats accepted the manual treatment without any behavioral indication of stress or pain, other than being more likely to defecate during or immediately following treatment. After 7 days, the experiment was ended and the adhesions were evaluated. The proximal stitch of the cecal hinge had avulsed in 3 controls and 7 treated rats (difference not significant by Fisher’s Exact test); these data were excluded.

**Fig 2:**
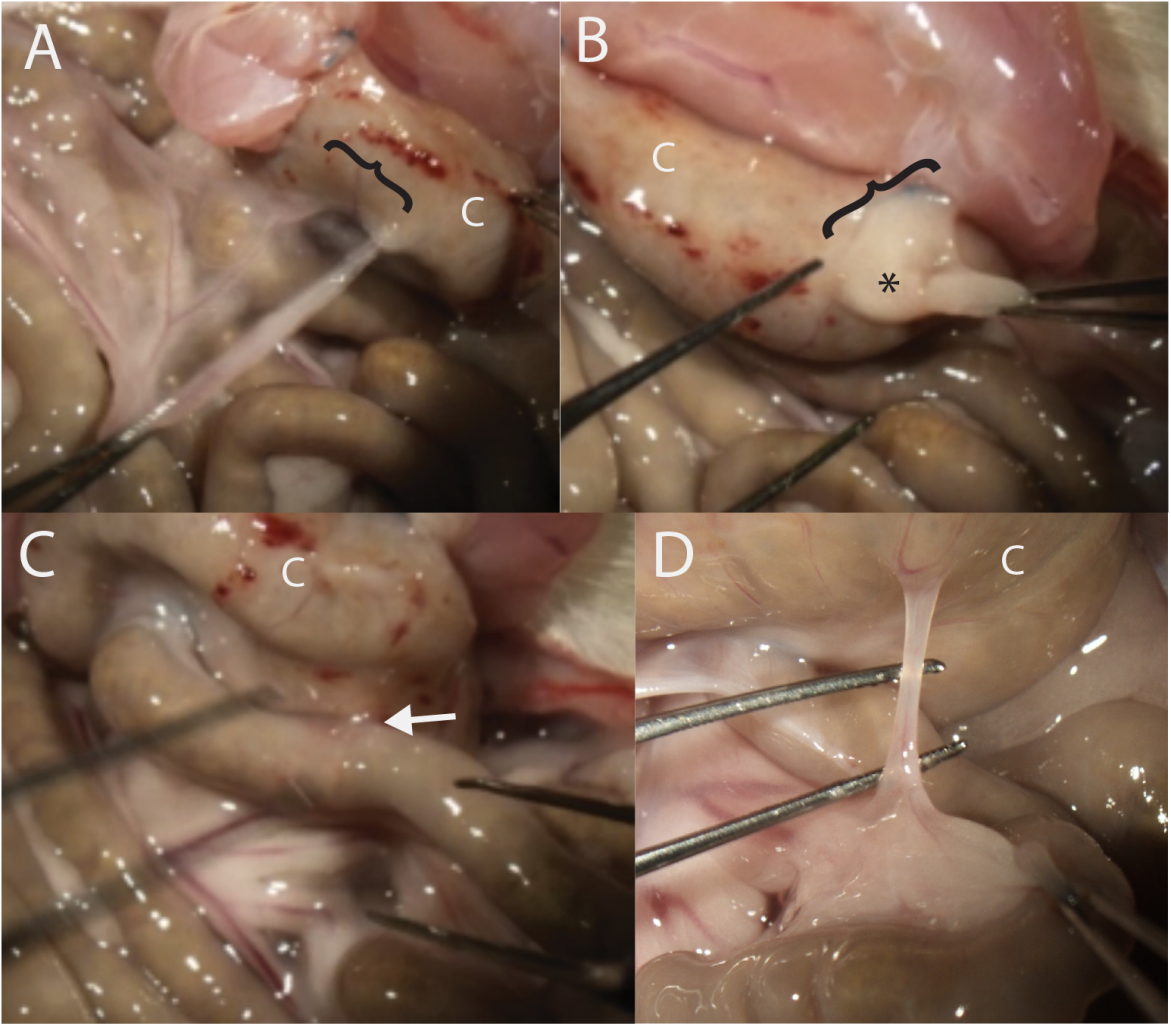
Representative postoperative adhesions seen in the cecal hinge model. Images A-C were taken from the same rat in the 4-day treatment group. A. Greater omentum to cecum (C, in all panels), the extent indicated by a bracket. B. Left adnexal fatpad (*) is adherent to the cecum. In this rat there was no primary adhesion, which can be appreciated by the fold in the tissue between the abdominal wall and the cecum. C. Cohesive adhesion between the small intestine and the cecum (arrow). D. Band-like adhesion between the mesentery of the small intestine and the cecum.

Besides the adhesion between the cecum and abdominal wall (Fig 1B), adhesions were usually found between the greater omentum and cecum, and adnexal fat pads and cecum. Adhesions connecting intestine to intestine (an example of a “cohesive adhesion;” [38]) were not found in all rats, but when they occurred they were flat, rather than band-like. Only 3 adhesions typically described as bands were seen in 143 necropsies, and these were between the liver and cecum (1), and between the small intestine and cecum (2). No adhesions were observed between non-cecal structures and the abdominal wall. Fig 2 shows representative images of these varied adhesions.

#### Primary adhesion areas

**Fig 3:**
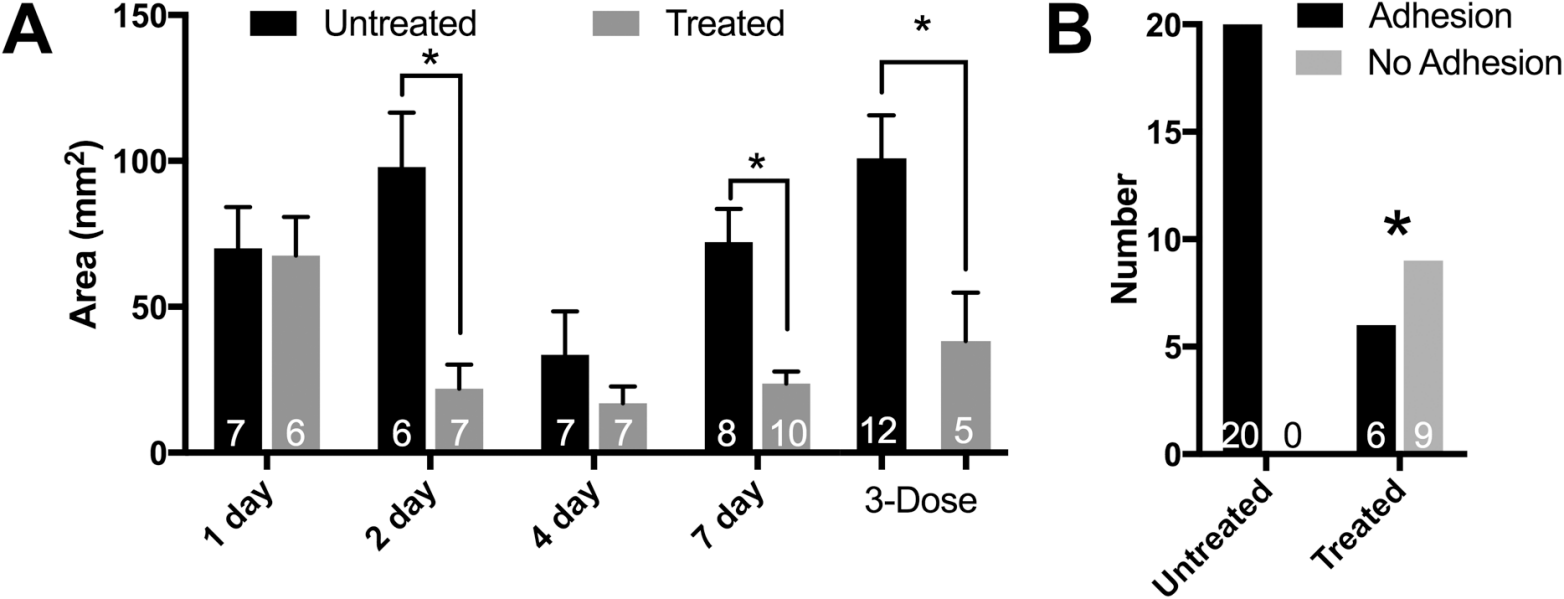
Modeled manual therapy attenuated or prevented primary postoperative adhesions. A. Areas of primary adhesions (means ± SEM; * p < 0.05). B. The proportion of rats with primary adhesion formation was lower than untreated rats (Fischer’s exact test, * = p < 0.0001). Numbers per group are indicated within bars.

The areas of 77 primary adhesions were measured and used for analysis. The primary adhesion areas were reduced due to the treatment (F_1, 51_ = 9.4, p = 0.0036) with post hoc tests showing significant differences at 2 and 7 days postoperatively (Fig 3A). The adhesion areas were also smaller with time (F_3, 51_ = 5.4, p = 0.0025). In the experiment where 3 doses of buprenorphine were given, we observed a significant decrease in the primary adhesion area with treatment (p < 0.05, Fig 3A). Although the mean size of the primary adhesions from the 3-dose untreated group was larger than the 1-dose untreated group, the difference was not statistically significant. Because there was no significant difference between the 1-dose and 3-dose groups, their outcomes were combined and analyzed for the presence or absence of primary adhesions. While all 20 rats in the 1-dose and 3-dose untreated groups had primary adhesions, only 6 of 15 rats in the treated groups had such adhesions, a statistically significant lower proportion (Fig 3B; Fischer’s exact test, p < 0.0001).

#### Adhesion ratings

**Fig 4:**
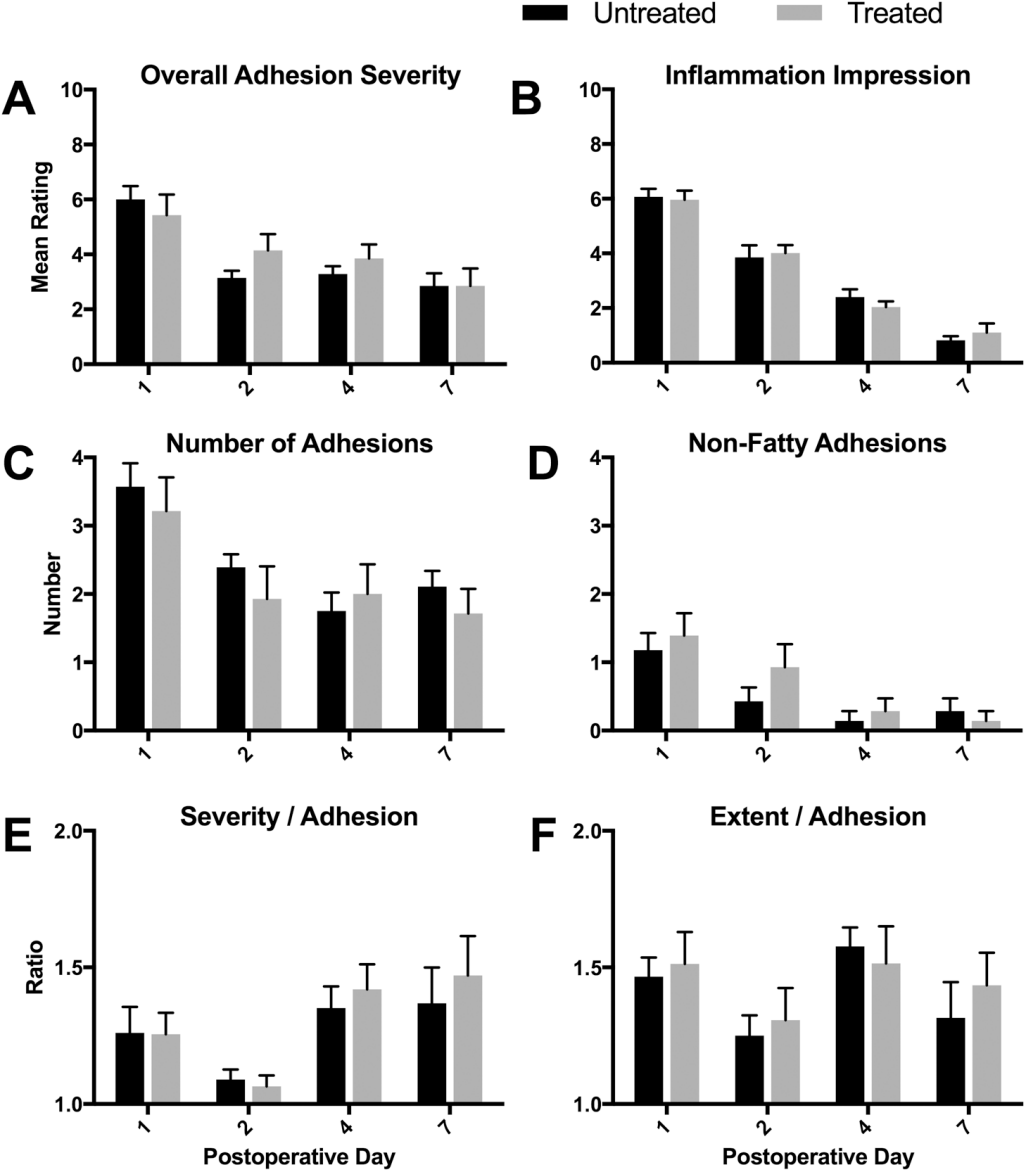
Ratings of necropsy videos (not including primary adhesion). There were no differences between treated and untreated groups in rated parameters of non-primary postoperative adhesions (n = 7 per group; means ± SEM). There were numerous differences over time, representing the natural biology of postoperative adhesions (Table 2).

Adhesions formed between the cecum and the greater omentum (Fig 2A) and the adnexal fat pads (Fig 2B), as well as less commonly between the cecum and the small or large intestine (Fig 2C) and the mesentery (Fig 2D). Ratings of these adhesions and the overall condition of the gut revealed no significant differences between treated and untreated rats in any of the measured parameters, which included overall severity, inflammation impression, number of adhesions, non-fatty adhesions (those not including omentum or fat pads), severity per adhesion, and extent per adhesion (Fig 4A–4F, respectively). When these data were analyzed by day, a number of differences appeared (Table 2). The overall severity decreased after the first day and then remained stable. The inflammation impression decreased significantly across all days for both groups. These observations are consistent with expected healing. The number of adhesions decreased consistently, and the severity/adhesion decreased at 2 days, but then increased. This is consistent with normal fibrinolytic activities, which increase after the first day, and then increases in strength as the fibrinous adhesions start to become fibrous.

**Table 2:** Statistical results for video ratings (Fig 4).

	Treatment		Day		Post-hoc <0.05	
Comparison	F (1,48)	p	F (3,48)	p	Untreated	Treated
(A) Overall Severity	0.46	ns	11.1	< 0.0001	1–2, 1–4, 1–7	1–7
(B) Inflammation Impression	<0.01	ns	105.2	<0.0001	all	all
(C) Number of Adhesions	0.87	ns	7.7	0.0003	1–4, 1–7	1–7
(D) Non-Fatty Adhesions	1.19	ns	9.59	<0.0001	1–4, 1–7	1–4, 1–7
(E) Severity / Adhesion	0.38	ns	5.46	0.002	none	2–7
(F) Extent / Adhesion	0.28	ns	2.47	ns	-	-

(A)–(F) refer to Fig 4. Statistically significant individual comparisons are indicated as numbers separated by hyphens, for example, “1–2” means that there was a significant difference between Day 1 and Day 2 data.

#### Fecal discharge

**Fig 5:**
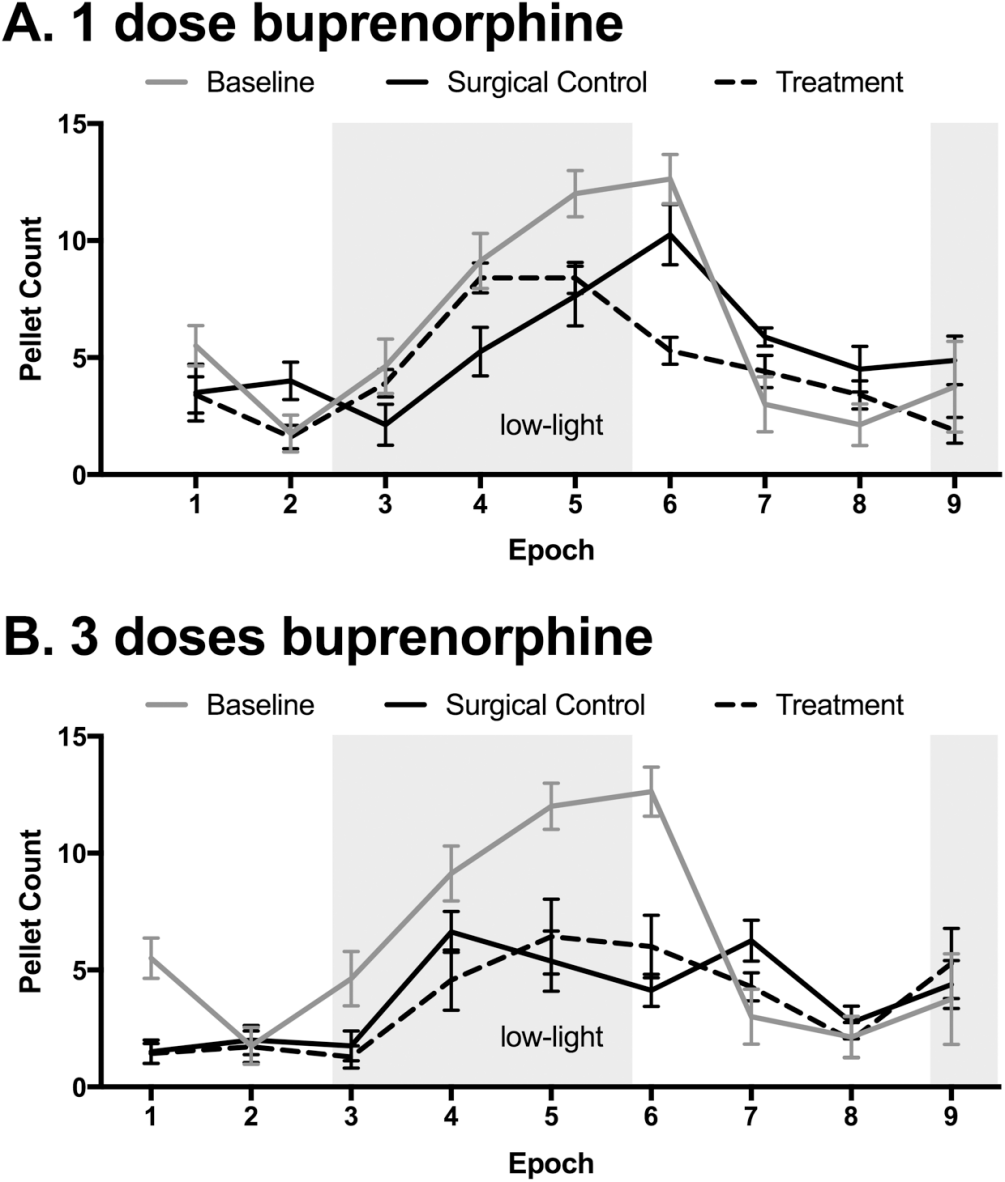
Effects of surgery and buprenorphine on fecal pellet discharge. A. Surgery and 1 dose of buprenorphine immediately after surgery (beginning of Epoch 1) led to reduced fecal pellet discharge 20 to 24 hours after administration. B. Surgery and 3 doses of buprenorphine immediately after surgery (beginning of Epoch 1) led to reduced fecal pellet discharge from 12 to 24 hours after administration. The observed differences between doses were not statistically significant, and there was no effect of treatment under either condition. Shaded area represents 7:00 PM– 7:00 AM. Baseline data in both panels are from rats from the 3-dose group, taken the week before surgeries. n = 14/group at each Epoch, means ± SEM.

Fecal pellet discharge was significantly lower following surgery (F_4, 576_ = 7.82, p <0.0001), but did not differ whether the rats had 1 or 3 doses of buprenorphine (0.05 mg/kg sc, Fig 5).

#### Intraperitoneal macrophage phenotype

**Fig 6:**
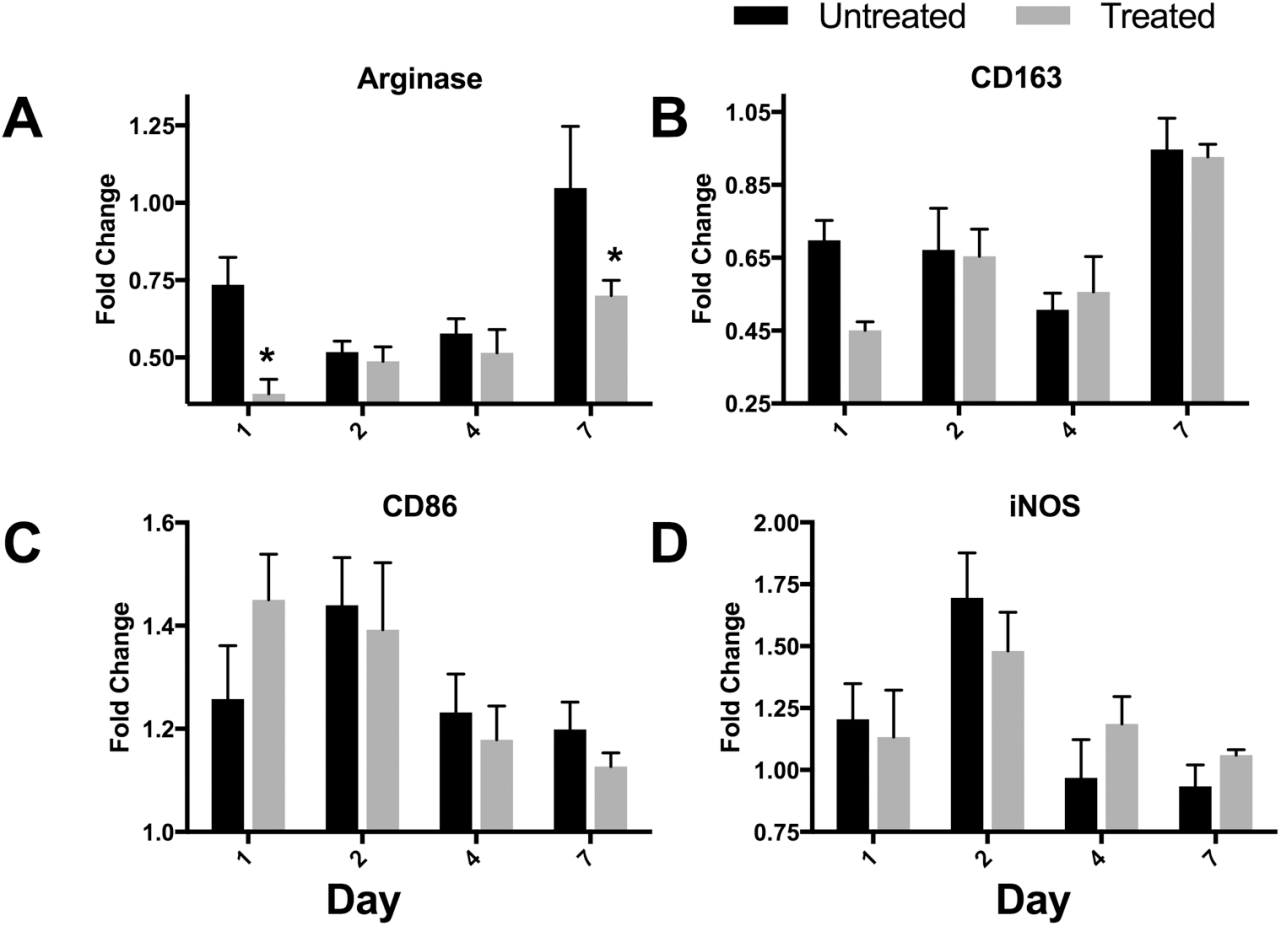
Relative expression of M1 or M2 markers by intraperitoneal macrophages. Intraperitoneal macrophages were isolated by peritoneal lavage at postoperative days specified in the X-axes. Cells were stained with anti-CD11b to positively identify monocyte lineage cells and anti-HIS48 to exclude neutrophils. Expression levels of the indicated markers (A) arginase, (B) CD163, (C) CD86, (D) iNOS are depicted as fold changes normalized to expression by intraperitoneal macrophages isolated from naïve rats. n = 7/group, means ± SEM. * p<0.05 post hoc test.

To determine if MMT had an effect on inflammatory status, we focused on intraperitoneal macrophages using the expression of inflammatory markers CD86 and iNOS and trophic markers arginase and CD163. Macrophage expression of arginase protein was reduced in the MMT rats (F_1, 50_ = 10.03, p = 0.0026), with post hoc tests showing significant differences at 1 and 7 days postoperatively (Fig 6A). Overall expression of CD163, CD86, and iNOS proteins did not differ by treatment (Fig 6B–6D). Expression of all markers differed by day [increased arginase (F_3, 50_ = 7.29, p = 0.0004); increased CD163 (F_3, 50_ = 11.68, p,0.0001); decreased CD86 (F_3, 50_ = 4.058, p = 0.012); and increased and then decreased iNOS (F_3, 50_ = 11.68, p = 0.0001)].

### Experiment 2. Modeled manual therapy following adhesiolysis

30 rats underwent a cecal sidewall surgery, and after 28 days underwent a second laparotomy for adhesiolysis. Of the rats in this study, 1 rat was found dead 2 days following the first surgery. Two control rats became moribund 3 and 4 days following the adhesiolysis, and necropsy determined that they had developed blockage of the large intestine. Results are from 12 control and 15 treated rats.

During adhesiolysis, all rats were noted to have substantial indurated fecal matter in their proximal ceca. The primary adhesions in the adhesiolysis surgeries required sharp dissection, and it was often difficult to distinguish cecum from abdominal wall. In most rats, 1 or more perforations were made in the cecum. These were stitched with 8–0 prolene and swabbed thoroughly. Other than the primary adhesions, the other adhesions within the abdominal cavity were comparable to those reported above, primarily involving the omentum and fat pads.

**Fig 7:**
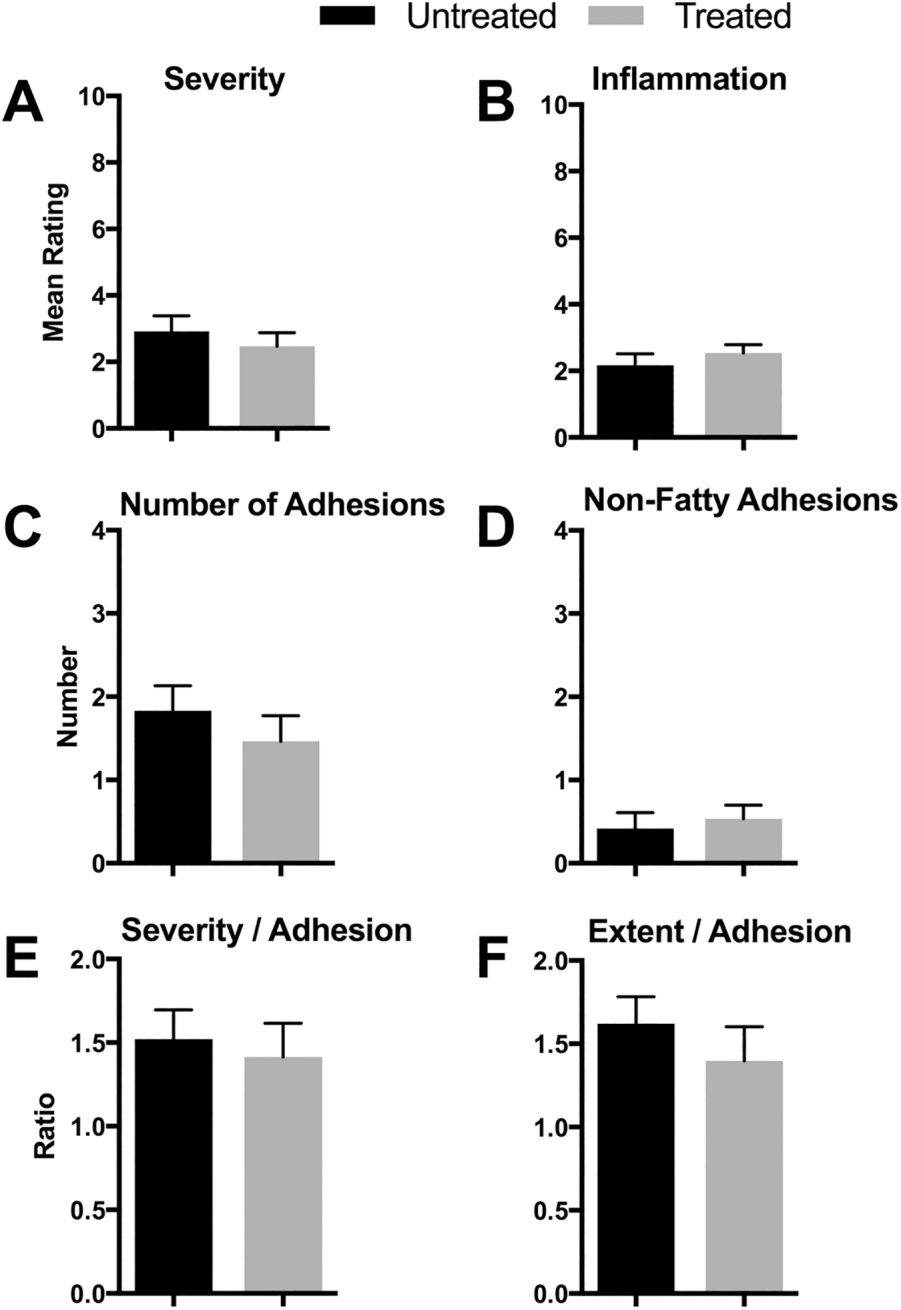
Ratings of necropsy videos 7 days following adhesiolysis. There were no differences between treated (n = 15) and untreated (n = 12) rats in adhesion parameters following adhesiolysis (means ± SEM). These results are comparable to those reported in Fig 4.

None of the 15 treated rats re-developed primary adhesions between the cecum and the abdominal wall, but 3 of the 12 untreated rats did. While a higher proportion, this difference was not statistically different (Fisher’s Exact test, p = 0.07). Ratings of the necropsies showed no difference between groups, and were almost identical to the ratings of the 7-day rats as reported earlier (Fig 7). This similarity is consistent with other reports of adhesiolysis in rats where there was no difference between the severities at initial versus post-adhesiolysis ratings [39–41].

### Experiment 3. Modeled manual therapy following strictureplasty

**Fig 8:**
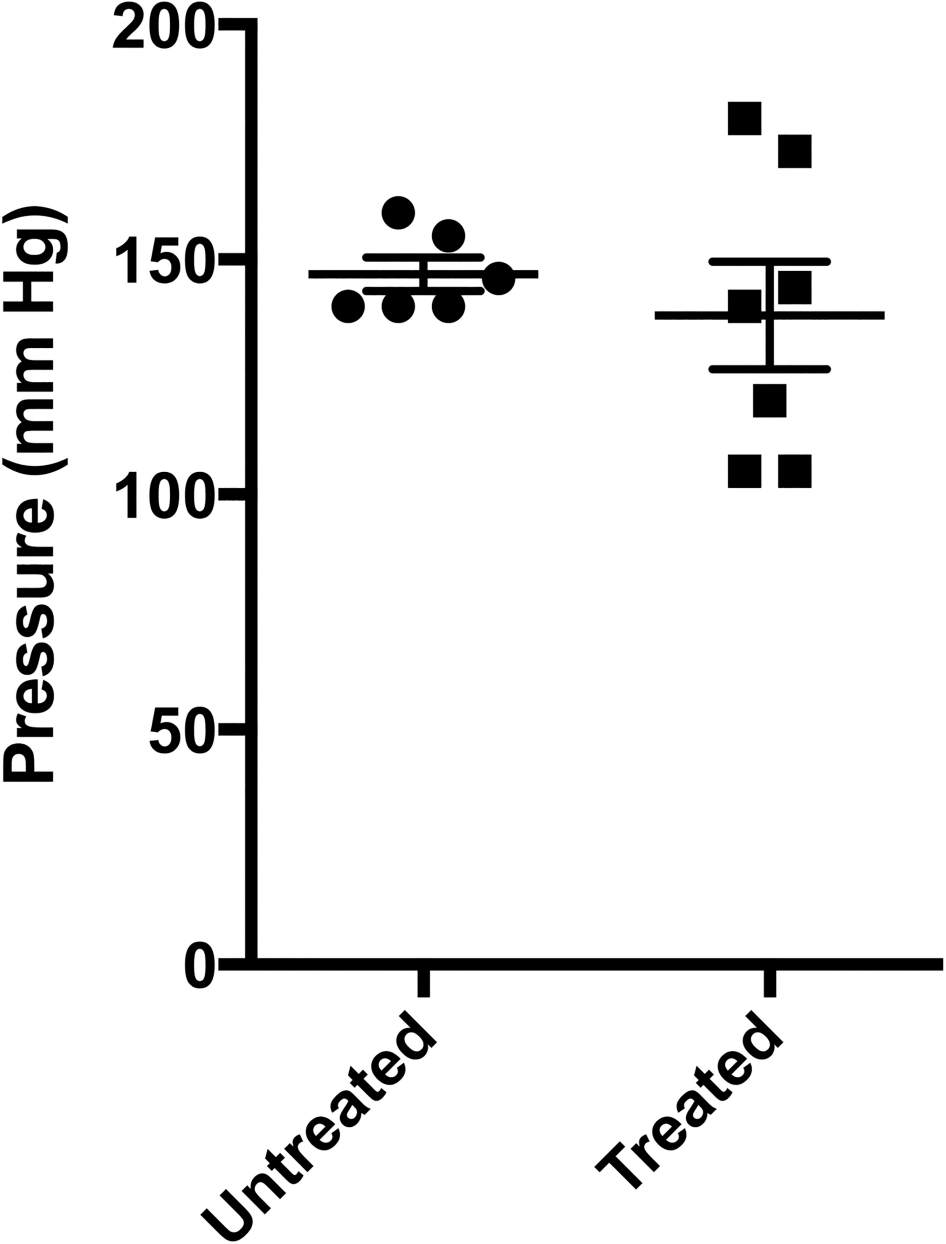
Strictureplasty burst strengths. While the variability of the burst strengths was greater following treatment (untreated n = 6, treated n = 7, means ± SEM), the means were not.

A strictureplasty (Fig 1C) was performed followed by MMT to evaluate the safety of the treatment on a surgery with very fine and presumably delicate suturing. There were no experimental losses due to the surgical procedure. The greater omentum was found to be adherent to the strictureplasty suture line in all 14 rats, and there was no apparent difference in the extent of these adhesions. There were no cohesive adhesions in any rats. There was no difference in the burst pressure between groups (Fig 8). As can be seen in the figure, the variability of the burst pressures was greater in the treated rats. However, in most tests, the mesothelium split at a lower pressure than the pressure that finally caused suture failure. This indicates that the pressures were all above physiological levels. The treatment did not augment or impair healing.

## Discussion

We have shown that applying MMT to the rat abdomen following an adhesion-inducing surgical procedure leads to smaller and less frequent cohesive adhesions between the cecum and the abdominal wall. Other cohesive and band-like adhesions were not prevalent enough in our cecal hinge model to reliably measure differences. Our treatment had no detectable affect on adhesions involving the greater omentum or adnexal fat pads in any experiments. Although the areas where these adhesions formed were treated, we attribute this observation to a relative lack of the ability to palpate and thus deliberately move the structures away from the cecum. Although there is no consensus, the literature supports that cohesive and band-like adhesions are more likely than thin or filmy adhesions to cause bowel obstructions and pain [42–45]. It remains unknown how thin and filmy adhesions in rats are relevant to the more substantial and fibrous adhesions in humans. On balance, our results support that MMT can be considered to have had a positive effect on adhesion prevention.

A therapist with extensive clinical experience designed our treatment approach to be a scaled down version of what is routinely used in manual therapy practice. Although we do not know if the mechanical effects of the treatment are similar between rats and humans, it is likely that the treatments induces similar sliding motions between structures. Therefore, we can expect that the treatment applied to humans should lead to attenuation of postoperative adhesions. One concern about mobilization immediately after surgery is that it could disrupt delicate sutures. However, we showed that in freely mobile surgeries (strictureplasties) that even very fine sutures were not disrupted by treatment directed to the repair site, and that healing was not impaired. It has been previously published that mechanical massage delivered to the abdomen using a hand held device immediately after abdominal surgery was found safe to apply and found effective in reducing postoperative ileus [46]. If our hypothesis is correct, that limiting postoperative ileus, or providing passive movements between structures until the mesothelium heals (3–4 days) will prevent postoperative adhesions between the anterior abdominal wall and the intestines, manual therapy deserves to be tested in humans for the potential reduction of postoperative adhesions.

We assayed postoperative digestive function as a measure of ileus using fecal pellet discharge. However, we did not measure the rats long enough to fully test our hypothesis. While the surgery and buprenorphine given at analgesic doses for 12–36 hours reduced fecal output, this reduction was not seen after 36 hours, and we observed that after two days the rat fecal output was back to normal. Our therapist (SLC) reported that on the 3^rd^ day of treatment there were no palpable changes of the adhesions and that the general “stickiness” of the abdominal contents was reduced, which is consistent with the reduced inflammation ratings reported in Fig 4B and the normal time course of fibrinolysis. Ideally, we would find a means by which to reduce postoperative gastrointestinal activity in the rat for up to 3–4 days, and would expect this to lead to worsened adhesions between the injured cecum and other structures. After this time, the mesothelium is becoming continuous (in rats as well as in humans), and further adhesions are not likely to develop. Moreover, we used young female rats, which are naturally more active than older rats, and used isoflurane anesthesia, which is very short acting and only reduces peristalsis during anesthesia. We also did nothing to limit activity of the rats, which resumed their normal activity patterns within an hour, possibly enhancing the effect of the MMT. All of these factors likely worked to limit the severity of our model, leaving less “room” to see effects of interventions, but they also emphasize that the MMT works beyond these factors. We hope in the future to more closely mimic postoperative ileus using drugs that restrict digestion and elimination for longer periods of time, and test the effects of drug interventions and activity levels, which vary by sex and decrease with age [47].

The primary mechanism of action of MMT could be quite simple. Peritoneal damage leads to fibrin secretion, and fibrin is an adhesive. Postoperative ileus may act like a clamp and thus allow the fibrin glue to “set.” The movement induced by MMT may simply keep the structures moving enough to prevent them from adhering until normal peristalsis resumes and fibrinolysis becomes dominant. Alternatively, MMT may disrupt fibrinous attachments prior to adhesion creation and/or newly created adhesions, when they are weakest. Our data also support that MMT has effects on intraperitoneal macrophages, which coordinate the inflammatory cascade, including healing. In untreated rats, CD86 and iNOS expression decreased over the course of the experiment, while arginase and CD163 increased in expression, particularly on Day 7. This profile is indicative of intraperitoneal macrophages that first instigate a primarily inflammatory response to the surgery that gradually switches to a predominantly trophic response by postoperative Day 7. In comparison, arginase expression was significantly lower in macrophages from treated animals, suggesting that the switch from an inflammatory to trophic response was delayed. From this we can conclude that the treatment inhibited the trophic macrophage response, and thus fibroblast activation, during the time when the mesothelium is regenerating. This potential mechanism of action for manual therapy, as well as an approach to limit postoperative adhesions pharmacologically, such as using safe prokinetic agents, is worth evaluating using macrophage functional assays in models of postoperative adhesions.

Although our previous study reported that in rat, adhesions could be prevented and disrupted [27], our more extensive experience is that while cohesive adhesions in rats can be palpated and treated, adhesions involving the omentum and fat pads cannot be palpated or treated. It has been suggested that manual therapy can reverse infertility, treat small bowel obstruction, and reduce chronic pelvic pain [48–51]. In these approaches, there is no knowledge of the type of adhesion towards which the treatment is directed, nor whether movement is being induced between the desired structures. It seems unlikely that any clinical effect of manual therapy would involve the reduction or lysis of cohesive adhesions, especially those that had become fibrous, which occurs within weeks of surgery. Since thin and filmy adhesions cannot be palpated in a rat, they may not be palpable in a human; even if not palpable, if MMT caused movement between the structures, and if our hypothesis is correct, these adhesions should be also attenuated in humans. If not palpable it seems unlikely that mature adhesions in humans could be disrupted by manual therapy. This does not imply that these treatments do not lead to reduced symptoms, but that there is likely a different mechanism at play.

Our results support that MMT initiated immediately postoperatively is an effective preventive for cohesive postoperative adhesions, which are the most problematic in humans. The techniques of visceral manipulation are not widely practiced, but learning to safely mobilize the postoperative abdomen is not complex. While there is a compelling possible mechanism related to macrophage phenotype, it seems more likely that the primary mechanism is the maintenance of relative motion between healing surfaces. If this is the case, it should not matter whether the intestines are passively moved using the hands or whether motility of the gut is maintained by other means, such as with tolerable prokinetic drugs or early ambulation. We hope to see further research directed towards the prevention of postoperative adhesions based upon these concepts.

## Supporting information

S1 Video This video is an example of the treatments performed. (MP4)
